# Aerobiology of the Family Lamiaceae: Novel Perspectives with Special Reference to Volatiles Emission

**DOI:** 10.3390/plants13121687

**Published:** 2024-06-18

**Authors:** Robert Adrian Haas, Ioana Crișan, Dan Vârban, Rodica Vârban

**Affiliations:** Department of Crop Science, Faculty of Agriculture, University of Agricultural Sciences and Veterinary Medicine of Cluj-Napoca, Calea Mănăștur Street No. 3-5, 400372 Cluj-Napoca, Romania; robert-adrian.haas@student.usamvcluj.ro (R.A.H.); dan.varban@usamvcluj.ro (D.V.); rodica.varban@usamvcluj.ro (R.V.)

**Keywords:** aromatic, phytochemistry, terpenoids, glandular trichome, pollinator, intercropping

## Abstract

Lamiaceae is a botanical family rich in aromatic species that are in high demand such as basil, lavender, mint, oregano, sage, and thyme. It has great economical, ecological, ethnobotanical, and floristic importance. The aim of this work is to provide an updated view on the aerobiology of species from the family Lamiaceae, with an emphasis on novelties and emerging applications. From the aerobiology point of view, the greatest interest in this botanical family is related to the volatile organic compounds emitted by the plants and, to a much lesser extent, their pollen. Research has shown that the major volatile organic compounds emitted by the plants from this botanical family are monoterpenes and sesquiterpenes. The most important monoterpenes reported across studies include α-pinene, β-pinene, 1,8-cineole, menthol, limonene, and γ-terpinene. Most reports tend to cover species from the subfamily Nepetoideae. Volatile oils are produced by glandular trichomes found on aerial organs. Based on general morphology, two main types are found in the family Lamiaceae, namely peltate and capitate trichomes. As a result of pollinator-mediated transfer of pollen, Lamiaceae species present a reduced number of stamens and quantity of pollen. This might explain the low probability of pollen presence in the air from these species. A preliminary synopsis of the experimental evidence presented in this work suggests that the interplay of the organic particles and molecules released by these plants and their environment could be leveraged for beneficial outcomes in agriculture and landscaping. Emerging reports propose their use for intercropping to ensure the success of fructification, increased yield of entomophilous crops, as well as in sensory gardens due to the therapeutic effect of volatiles.

## 1. Introduction

Aerobiology is a field of science that studies particles of biological origin that are small enough to travel with air masses and air currents [1]. Both the outdoor atmospheric domain [2] as well as the indoor environment [3] are studied within this science. This includes several topics that intersect with many other sciences. The research area of agricultural and biological sciences has consistently dominated the aerobiology field in the last decades, demonstrating the increasing practical interest in the subject, followed by the areas of medicine, immunology, and microbiology, among other minor domains [4]. Such organic particles are omnipresent in the air and can have their origin in different sources, such as the plant canopy, soil, water surface, and anthropic environments [5]. The air-borne biological particles studied by aerobiology, known as bioaerosols, can be either whole organisms or fragments from plants, fungi, protists, prokaryotes, and viruses, as well as minute animals such as small insects [6]. The products emitted by these, such as toxins [7] or biogenic volatiles, are also subjects of this field [8,9]. Although their presence in air masses can be more or less dispersed, these particles can travel large distances and influence atmospheric processes, interfere with other living systems, and cause observable biological or ecological effects [5].

The biological contribution of plants to ambient air has complex outcomes. It has been shown that vegetation enriches the atmosphere with organic aerosols, playing an important macro-scale role in atmosphere–biosphere feedback. The biogenic volatile organic compounds (BVOCs) emitted by such vegetation can constitute condensation nuclei that are able to form clouds; furthermore, they could contribute to the formation of bright clouds that better scatter sunlight, resulting in a cooling effect [10]. In natural and human-managed ecosystems, plants release volatile organic compounds which facilitate outcomes derived either directly or indirectly from their attractant or repellent effect on other organisms. These compounds have roles as signaling molecules in plant defense, pollination, and inter-plant communication [11]. In indoor spaces, plants are known for improving air quality by decreasing the suspended potentially pathogenic microorganism load [12] and releasing negatively charged ions beneficial for human health [13]. The particulate contribution of plants to air has been well studied, especially with regard to the allergenic potential of pollen [14]. For example, a previous study created a useful aerobiological risk map for the family Cupressaceae in the Iberian Peninsula for the safe planning of outdoor activities and to prevent pollen allergy cases [15]. Some studies have been concerned with the aerobiology of a single plant species in order to elucidate certain pollination mechanisms, such as in *Symplocarpus foetidus* [16]. The interplay of the organic particles and molecules released by the plants and their environment suggests that at least some could be deliberately leveraged for beneficial results. The cultivated or widespread plants known to be rich in volatiles could be of particular practical relevance.

Currently, the works dedicated to the aerobiology of a given botanic family are scarce; hence, this work intends to identify the relevant aerobiological aspects for one of the botanical families most rich in volatiles—Lamiaceae. This work further aims to highlight the importance of gathering information on a botanic family in a holistic way, with the purpose of discovering unexplored applications and identifying gaps in knowledge. This work assembles the relevant information to create a new perspective on this family. In this paper, we invite readers to gain an overview of the family Lamiaceae from the perspective of aerobiology and thus have a novel understanding of the species from this botanical family, from botany to practical implications.

Lamiaceae is a large family of flowering plants, which is rich in aromatic species. It holds great importance economically, ecologically, ethnobotanically, and floristically [17,18]. It is widespread in natural habitats, while the growth of crops is expanding as a result of high demand [19,20]. Hence, aromatic Lamiaceae species could become an important presence, as well as a potential organic aerosol contributor via their produced volatiles. The volatiles’ multiple interactions with other groups of organisms, from microorganisms (including potentially pathogenic ones) to other plants, animals (especially insects, herbivores, and pets), as well as humans, deserve a better understanding.

Given the huge economic importance of this botanical family, it is understandable why the specialist literature is exceptionally consistent on various research topics for species from Lamiaceae. Yet, to our knowledge, there is no overview that gathers the relevant findings on the aerobiology of this family, despite its widespread presence, cultivation, and use.

The aim of this work is to provide a general view on the aerobiology of the family Lamiaceae. This work considers the main aspects of aerobiology, such as volatiles and pollen, with the purpose of clarifying their relevance and placing an emphasis on emerging aspects with potential practical implications. This work seeks to gather examples on the commonly cultivated species of Lamiaceae due to their relevance. The goal is to provide an initial synopsis of the current knowledge by bringing attention to an approach that can be useful to better identify the unexplored potential of these species.

## 2. Botany Aspects of the Family Lamiaceae

### 2.1. Plant Morphology

The family Lamiaceae comprises about 3500–4000 species of plants largely spread throughout the warm and temperate Holarctic region. The vast majority of these plants are annual or perennial herbs, but there are also shrubs. The stems are quadrangular, with the leaves arranged opposite and decussate. The leaves are more often simple and margin entire to divided. Flowers are zygomorphic, with a characteristic bilabiate morphology, and are arranged in axillary cymes or in terminal spikes. The calyx is symsepalous and the corolla sympetalous. The stamens are frequently four and didynamous (only two stamens in the genus *Salvia*), often with a specialization for entomophilous pollination. The two carpels have a superior ovary and gynobasic style. The nectaries are present at the base of the ovary. The schizocarp fruit consists of four nutlets [21]. The flower morphology of the Lamiaceae species favors cross-pollination [22].

There are several high-diversity regions in the world. The family Lamiaceae remains particularly well represented in the Mediterranean region and Southwest Asia [23]. Greece reports the presence of 414 taxa of Lamiaceae in the country, including 111 species endemic to Greece [24]. Turkey reports 48 genera and 603 species, out of which 271 are endemic [25]. The flora of China reports 96 genera and 807 species [26]. In Mexico, 32 genera have been reported, comprising 591 species, notably with a high endemism rate of 65.82% [27]. In South Africa, there are 42 genera with 297 species, of which 105 are endemic [28]. In the Neotropics, the family is represented by about 65 genera and 1,690 species [29]. Olmstead check listed over 20 genera distributed in Australia, with several of them native [30].

Most people are familiar with certain species of this botanic family due to their frequent use as culinary herbs (*Mentha* × *piperita*, *Salvia rosmarinus*, *Ocimum basilicum*, *Origanum majorana*, *Origanum vulgare*) [31], valuable medicinal plants (*Salvia officinalis*, *Melissa officinalis*, *Thymus serpyllum*) [32], or highly melliferous species (*Dracocephalum moldavica*, *Lavandula angustifolia*, *Leonurus cardiaca*, *Salvia verticillata*) [33]. Some species are appreciated as ornamentals due to their flowers (*Salvia splendens*) [34] or their fruits (*Callicarpa* sp.) [35], while others confer emblematic notes in the fragrance industry (*Pogostemon cablin*) [36]. Many species are sources of aromatic oils that possess bioactivities (*Agastache rugosa*, *Lavandula angustifolia*, *Dracocephalum officinalis*, *Satureja hortensis*, *Betonica officinalis*, *Thymus vulgaris*) [37], while others are known for their strongly psychoactive effects (*Salvia divinorum*) [38]. The species that are important due to their uses are also cultivated over larger areas. Also, many of their applications are documented in relation to the essential oils extracted from these species [39]. The volatiles that are naturally emitted by these plants in the environment also deserve attention with regard to their potential role.

### 2.2. Pollen Grains

In the Lamiaceae family, the evolution of bilabiate flowers caused a reduction in stamen number, which is further associated with decreasing pollen production and increasing the precision of pollen transfer mechanisms [40]. Both the entomophilous and ornithophilous pollination types have been reported in this botanic family, with pollinators acting as carriers of pollen from one flower to another. Pollinators observed for some common Lamiaceae species belong to Hymenoptera, Diptera, and day-flying Lepidoptera [22]. Birds and beetles have been reported as well, such as in the *Prostanthera* genus in Australia [41]. Other interesting pollination mechanisms have been reported, such as explosive release of pollen in the ornithophilous species *Hyptis pauliana* from Brazil [42].

The pollen in Lamiaceae is of the colpate type [43]. The morphological characterization of pollen is based on variation in size and shape, on exine ornamentation pattern, colpi, number of perforations, occurrence of large central secondary lumina per primary lumen, as well as the number of secondary lumina per primary lumen. All these hold taxonomic significance and still provide taxonomic insights, as demonstrated in recent studies conducted on *Salvia* [44], *Clerodendrum* [45], and *Nepeta* [46]. The nutritional value of Lamiaceae pollen recommends their use, particularly for improving the food resources of hive bees [47].

Aeropalynology is a major chapter in the domain of aerobiology. However, our literature screening did not return many results, indicating that species of this family are not important contributors of pollen to the air, a fact that might explain the low number of reports and studies. A study conducted in the urban area of Canakkale, Turkey, reported a low degree of pollen allergenicity for Lamiaceae and a low average for total pollen counts of Lamiaceae taxa [48]. A study conducted in Poland on the variation in airborne herbaceous pollen from urban and rural areas showed the seasonal pollen index (SPI) of Lamiaceae was higher in rural than in urban areas. The SPI values ranged from 3 to 22 for Lamiaceae, which were overall lower than those reported for other entomophilous families such as Asteraceae, yet within close range of the values registered by Ranunculaceae [49]. Another study conducted in the Nam Co Basin in central Tibet showed that, while annually the herbaceous pollen represented 87.03% of the total sum of pollen in that type of habitat, the Lamiaceae pollen represented merely 0.14% [50]. An aero-palynological study from southwestern Nigeria confirmed Lamiaceae pollen presence from *Hyptis suaveolens* during three months of the year, as well as pollen from *Ocimum gratissimum* during eight months of the year [51], indicating that while pollen contribution to ambient air was low, it was possible to detect a species seasonality.

The modest number of reports could be explained by the low number of stamens in these species. Pollinators act as carriers of pollen on their backs or beaks; hence, this specialization might explain a low probability of pollen presence in the air. This is in comparison to anemophilous plant species of other botanic families that release large amounts of pollen in the air to be carried by the wind. The aerobiology studies do not abound in reports of Lamiaceae pollen, and, as a consequence, their relevance for this field of science remains low. This is in contrast with the volatile organic compounds emitted by these plants, which are of much greater interest for aerobiology, as will be presented in the following chapters.

### 2.3. Trichomes—The Structures Emitting Volatiles

Plants have dedicated organs and tissues specialized to produce and store metabolites, which are located internally or on the surface. The second category is broadly represented by glandular trichomes [52]. The glandular trichomes are found on the aerial organs of about 20–30% of vascular plant species [53]. These play a modulating and defensive role for the plant in relation to biotic and abiotic environmental factors, while the secretion products are gaining practical importance. Moreover, precision breeding by genome editing with the purpose of creating genotypes that display customized glandular trichome parameters is one possibility to harness their benefits [54].

**Figure 1:**
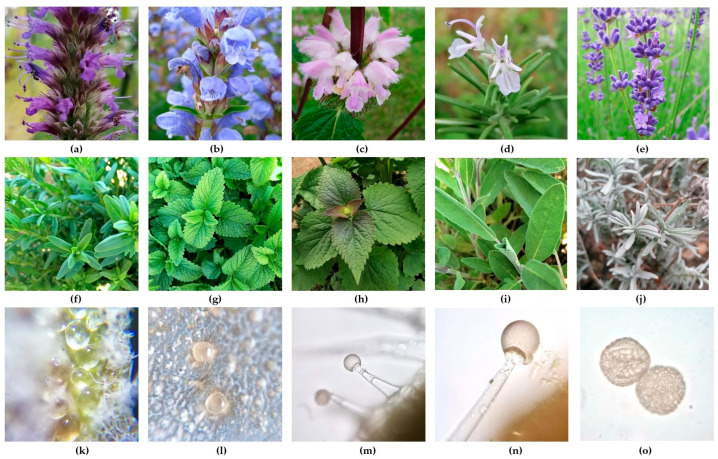
Morphological details of some Lamiaceae species: inflorescence of *Agastache foeniculum* (**a**), *Dracocephalum ruyschiana* flowers (**b**), *Phlomoides tuberosa* flowers (**c**), *Salvia rosmarinus* flowers (**d**), *Lavandula* × *intermedia* flowers (**e**), *Dracocephalum officinalis* leaves (**f**), *Melissa officinalis* leaves (**g**), *Agastache rugosa* leaves (**h**), *Salvia officinalis* leaves (**i**), *Lavandula angustifolia* leaves (**j**), large peltate trichomes on calyx of *Lavandula angustifolia* (**k**), short-stalked capitate trichomes with four-celled heads on *Ocimum basilicum* leaf (**l**), long-stalked glandular trichomes on *Salvia sclarea* leaf (**m**,**n**), pollen grains of *Ocimum basilicum* (**o**) (original photos by I.C.).

The epidermal structures (indumentum) of the family Lamiaceae comprise both glandular and non-glandular trichomes [55], distributed on vegetative and reproductive organs. The morphology of glandular trichomes reported in this family varies [56,57]. Based on general morphology, two main types have been described in the Lamiaceae: peltate trichomes with a disk-shaped head and very short stalk and capitate trichomes with a longer stalk and a head comprising one to several cells. The glandular trichome has a base cell that connects it to the plant surface. The stalk cells present large vacuoles, and the neck cell has cutinized walls to prevent the backflow of secreted substances. The secretory head cells have a dense cytoplasm with leucoplasts. Through the separation of the outer cell wall and cuticle of the head, a space is formed where the secreted metabolites are accumulated. When the cuticle is broken, the secretory products from the subcuticular space are released. In valuable aromatic Lamiaceae species, these secretion products are represented by precious volatiles rich in monoterpenes [53]. Raffinose-based sugars are predominant in the phloem of the family Lamiaceae and represent the source of carbon for glandular trichomes [52]. In the biosynthesis pathway of monoterpenes in glandular trichomes of species from the Lamiaceae, several enzyme classes are involved, such as monoterpene synthase, CYPs, or reductases [58]. The European Pharmacopoeia (EP) refers to the peltate glandular trichomes found in the species from the Lamiaceae family as “secretory trichomes of lamiaceous type”. The morphology of glandular trichomes is detailed in the EP for several medicinal species of Lamiaceae [59]. There is a relationship between the peltate trichomes and the quantity of volatile oil produced in the aromatic members of the Nepetoideae. Hence, these can be considered the most valuable structures to study in relation to volatile emission. They appear under a microscope, regularly distributed over the surface, located in small depressions, and varying in color from yellow, white, to transparent [60]. Some morphological aspects are presented in Figure 1.

A study on the leaf and stem of several oil-bearing species from the Lamiaceae, which are of interest for several types of industries, reported large peltate trichomes in *Mentha* × *piperita* (70.61 μm in diameter) and the smallest in *Mellissa officinalis* (44.26 μm in diameter) [61]. A comparative study of lavender cultivars has provided evidence that peltate trichomes are the largest on the calyx, with a diameter exceeding 100 μm. Due to this, the flowers were shown to be the main sites where VOCs are accumulated in this widespread cultivated species [62]. Because the nectar itself does not attract pollinators, it is supposed that glandular trichomes present on various parts of the flower play a double role through the volatiles they emit. At first, they provide protection for the buds by exerting a repellent effect, but after the flowers open, they have a role in attracting pollinators. In this regard, in *Origanum syriacum* and *Origanum vulgare*, capitate glandular trichomes on the corolla lobes were observed [63].

## 3. Volatile Emission of the Family Lamiaceae

The phytochemistry of the family is complex and diverse. Yet, two main groups can be distinguished within this family. The first group includes all species producing volatile terpenoids (notably monoterpenes, sesquiterpenes, and diterpenes) which therefore are essential oil-producing plants from the subfamily Nepetoideae. The second group comprises species that produce mostly non-volatile secondary metabolites in the polar fraction and are less valuable for essential oil production, including members of the subfamilies as follows: Ajugoideae, Callicarpoideae, Lamioideae, Peronematoideae, Premnoideae, Prostantheroideae, Scutellarioideae, and Viticoideae. However, the polar fraction metabolites can serve as chemotaxonomic markers at any level from species to subfamily and encompass seven classes of compounds, namely non-volatile terpenoids, lignans, flavonoids, iridoids, phenyl-ethanoid glycosides, caffeoyl-quinic acids, and phenolic acids. The rest of the subfamilies (Cymaroideae, Symphorematoideae, and Tectonoideae) cannot be currently assigned to either of the two groups, as more work on their phytochemistry is needed [55].

Plants can release volatile organic compounds (VOCs) from various parts of their organs, such as leaves, flowers, roots [64], and fruits [65]. They represent around 1% of the total secondary metabolites of plants [66] and play a crucial role in a wide range of physiological and ecological interactions. VOCs are one of the most important attractant signals for pollinators and are often surprisingly specific [67]. In general, for pollinators such as honey bees (*Apis mellifera* L., family Apidae, order Hymenoptera, class Insecta), the scent of plant volatiles carried by returning bees on their bodies becomes a source of chemical information for hive mates and is related to information about available floral resources [68]. The VOC emission regulates key ecological functions, being involved in plants’ protection against abiotic stressors such as temperature and light, by acting as antioxidants and cell membrane stabilizers [69]. Also, VOCs play an important role in natural seed dispersal [70,71,72], and they act directly or indirectly as protection mechanisms against herbivores [73,74] and pathogens [75]. VOCs also mediate intraspecific inter-plant communication [76].

Plant VOCs are small molecules with low molecular mass (100–500 Da) and high vapor pressure (0.01 kPa or higher at 20 °C) that quickly evaporate into the atmosphere [77]. These compounds are largely lipophilic products that belong to the chemical categories of terpenes and non-terpene aliphatics (including nitrogen- and sulfur-containing compounds), C5-branched compounds, phenylpropanoids, and benzenoids [11]. Many of these categories of compounds have been found in most orders of flowering plants, but 12 compounds are frequent, with the monoterpene category being by far the most present. Compounds (monoterpenes) occurring in more than half of the families of seed plants are limonene (71%), (E)-ocimene (71%), myrcene (70%), linalool (70%), α-pinene (67%), and β-pinene (59%) [78].

Headspace solid-phase microextraction sampling techniques showed that many flowering plants produce their most varied mixture, with the highest abundance of VOCs usually from their flowers [78]. However, in aromatic and medicinal plants (including the Lamiaceae family), the leaves also contribute significantly to VOC emissions. The essential oil and volatile profile (volatile emission) of Lamiaceae species is notably rich in volatile monoterpenes, sesquiterpenes, and diterpenes. The most important monoterpenes are α-pinene, β-pinene, 1,8-cineole, menthol, limonene, and γ-terpinene [79]. These monoterpenes occur in more than half of the families of seed plants [78]; therefore, the distribution of emitted volatiles in Lamiaceae family plants is not within phylogenetic limitations. Many of the main compounds found in the volatile profile obtained from Lamiaceae species (with some exceptions) are not reliable chemotaxonomic markers because they have been reported in other botanic families as well, and their accumulation can vary depending on environmental factors. Hence, in the family Lamiaceae, chemotaxonomic importance remains mostly attached to the polar fraction metabolites, not the volatiles [55].

Terpenes are largely found as constituents of volatiles, being mostly hydrocarbons. Terpene hydrocarbons have a molecular formula of (C_5_H_8_)*n*, where the *n* dictates the number of units involved as follows: monoterpenes—2 isoprene units, 10 carbon atoms; sesquiterpenes—3 isoprene units, 15 carbon atoms; diterpenes—4 isoprene units, 20 carbon atoms; triterpenes—6 isoprene units, 30 carbon atoms; tetraterpenes—8 isoprene units, 40 carbon atoms [80]. Terpenes are typically volatile, while terpenoids may be non-volatile or semi-volatile as they normally contain other polar moieties. In addition to terpenes, the volatile profile of plants in the Lamiaceae contains a few other chemical compounds. In Table 1, the most important volatiles identified among Lamiaceae family representatives are presented.

The above table (Table 1) summarizes findings from studies in which headspace solid-phase microextraction was used to collect the volatiles emitted by a fresh plant. The reason for choosing this method is to target only the volatile components that are actually released into the atmosphere. Studies that used extractive methods (e.g., distillation) were not considered here since it might be possible that the compounds detected in plants by other methods are not always released from flowers or leaves in natural settings. Headspace solid-phase microextraction is an equilibrium methodology that requires the prior modification of experimental parameters in order to maximize the extraction efficiency of volatile metabolites and thus acquire the most precise volatile profile [129].

The dominant categories of VOC emission in the family Lamiaceae were the monoterpenes (MHs and OMs) and sesquiterpenes (SHs predominantly and less frequently OSs). Diterpenes and triterpenes are less volatile [130], as well as less abundant than mono- and sesquiterpenes; therefore, they are rarely seen as significant components in volatile emission from fresh plants.

Also, 2,5-dimethyl-3-methoxypyrazine was identified as a major compound in the volatile emission of fresh leaves of *Mentha spicata* [66], representing one of seven metoxypyrazines also identified in grapes or wine [131]. Several non-terpene derivates are major compounds in Lamiaceae representatives: 1-octen-3-ol specific to *Melittis melissophyllum* subsp. *melissophyllum* [98], as well as fatty acids and fatty acid esters specific to *Salvia* sp. [109], *Mentha* sp. [100], and *Scutellaria* sp. [119].

As for the scent of individual compounds, it has been reviewed by Chizzola in terms of sensory characteristics perceived by humans. Among these, camphor is described as fresh-ethereal, limonene as fresh citrus-like, myrcene as mild-sweet and balsamic, linalool as floral, α-pinene as sharp-woody, β-pinene as spicy, β-caryophyllene as woody-spicy, and germacrene D as fruity [132]. However, pollinators detect these volatiles with their antennae, and for them, these compounds represent important information signals that guide their decisions and flight patterns. Perception and function of olfactory pathways in insects can be measured through electro-antennography, which determines the neuronal electric signal of the antenna for a given molecule. Around 30% of current crops depend on successful pollination for a fruit and seed set; volatile emission is particularly relevant from this perspective. Among pollinators, *Apis* sp. remains one of the most important for agriculture due to its manageable, large colonies that can be easily deployed in the vicinity of crops to ensure successful yields. Hence, honey bee behavior in relation to volatile emissions from plants has enjoyed higher interest compared to that of other pollinators. Bees associate some of these volatile compounds with food rewards. But it has also been shown that bees learn to distinguish their signals, with variable memory depending on age and season [68].

## 4. Practical Implications of the Volatiles Emission

The rich, volatile profile of some Lamiaceae has some novel aerobiology-related practical implications. Monoterpenes (MHs and OMs), identified as the dominant category in volatiles emission of Lamiaceae representatives (Table 1), consist of a veritable weapon against pathogens. Monoterpenes can act against field crop insect pests in many ways, having important practical implications for intercropping. They can also be botanical pesticides and serve as lead candidates for the development and synthesis of novel monoterpenoid pesticides for agricultural use. Monoterpene modes of action are as follows: contact toxicity, fumigant toxicity, deterrent and antifeedant effects, growth inhibitory effects, and residual toxicity [133]. Linalool, myrcene, limonene, thymol, 1,8-cineol, and α-pinene, to name only a few monoterpenes, can act on the nervous system or on the cell membranes of crop pests (putative mode of action) [134]. The compound 3-hexenyl acetate, a volatile reported in *Lamium* species (*Lamium amplexicaule*, *Lamium purpureum*) (Table 1), belongs to “green leaf volatiles” (GLVs), molecules that intervene in plant defense to abiotic attack [135]. These compounds were shown to be involved in recruiting parasitoids or carnivorous predators into repelling or disposing of herbivorous arthropods [136]. Green leaf volatiles are almost ubiquitously produced by green plants. The compound 1-octen-3-ol, specific to *Melittis melissophyllum* subsp. *melissophyllum*, is responsible for the distinct fungal scent and flavor of edible mushrooms and has an ecological role in attracting flies and mosquitoes, which are helpful in the reproduction process or as a defense mechanism [98].

There is a revived interest in the practice of intercropping, which opens interesting opportunities for the sustainable intensification of agriculture. Mixed cultivation provides crop diversification with potential benefits derived from lower inputs required and enhanced yields [137]. Further down is compiled an overview of some of the most relevant examples for species from the Lamiaceae family, with regard to the effects of interest that were attributed to the volatiles emitted by the fresh plants. These recent examples place emphasis on agricultural settings.

The experimental evidence suggests that aromatic plants can be successfully used to control pests in some crops due to the repellent effect of their volatiles. The species *Satureja hortensis* and *Ocimum basilicum* demonstrated a repellent effect against black bean aphids (*Aphis fabae* Scopoli, family Aphididae, order Hemiptera, class Insecta). The mechanisms at play rely on the volatiles released by the aromatic plants, which are non-hosts for the aphids. Due to this effect, aromatic plants have the ability to decrease aphid infestation when planted near target crops [138]. The polyphagous onion thrips (*Thrips tabaci* Lindeman, family Thripidae, order Thysanoptera, class Insecta) are reportedly repelled by *Lavandula angustifolia*, *Ocimum gratissimum*, *Origanum majorana*, *Mentha arvensis*, and *Salvia rosmarinus*. This presents the possibility of using these plants for integrated pest management. It was proposed that aromatic plants might be effective in controlling a wider range of Thysanoptera species that affect crops [139]. A study demonstrated statistically significant repellent effect of volatiles from *Ocimum basilicum* against the green peach aphid (*Myzus persicae* Sulzer, family Aphididae, order Hemiptera, class Insecta). This further underlines the potential use of some aromatic companion plants as an eco-friendly strategy to optimize pest management in orchards, but not only [140]. Alternating rows or alternating plants of *Amaranthus cruentus* with *Ocimum gratissimum* showed a beneficial effect on amaranth yield and repelled the pests specific to this crop [141]. *Capsicum frutescens* showed increased productivity parameters and decreased pest attacks when associated with *Ocimum basilicum* [142]. VOCs emitted by *Mentha* × *piperita* were shown to reduce infestations with the fruit fly (*Drosophila suzukii* Matsumura, family Drosophilidae, order Diptera, class Insecta), a devastating pest of berry fruit crops. Hence, it was proposed that intercropping and mowing the peppermint during fruit ripening stages might decrease infestation as large amounts of VOCs are released in the air [143]. Because these pests are also disease vectors, it might be possible that by reducing pest infestation with the help of volatiles produced by aromatic plants, disease incidence will also decrease. In this regard, further research could provide valuable insights. Based on these promising results, it is worthwhile to further investigate the potential of aromatic plants from the Lamiaceae family because they could provide natural plant protection. This is of current importance considering the justified interest in reducing pesticide applications due to health concerns.

Flower strips are used in agriculture to improve pollination. This is particularly important for seed production in pollinator-dependent crops. Melliferous aromatic crops could act as an attractive spot for pollinators. In this regard, an experiment showed that bee visitation to sunflower heads increased due to flower strips containing 12 melliferous species, including *Salvia pratensis* [144]. In intensive agricultural areas, ensuring flower strips and semi-natural habitats (which include Lamiaceae species) in the vicinity of crops with the purpose of creating a refuge and attraction point for bees can result in more visits to the flowers of the nearby crops, therefore increasing seed yield—as demonstrated for sunflower crops [144,145]. A meta-analysis provided increasing evidence that flower strips improve pest control and seed yield in the adjacent fields [146], indicating that this is a justified path of research. Given the highly simplified landscapes of intensive agricultural areas, it is advised to use flower strips or intercrop aromatic species with various crop plants to attract pollinators and benefit from a natural pest control mediated by VOCs from the air that are emitted by the aromatic plants.

Novel and less explored are the allelopathic effects of volatiles released in the air by plants. Allelopathic properties have been identified so far in some species from Lamiaceae, with several classes of compounds produced by these plants being potentially responsible for the inhibitory effect on other plants or, on the contrary, exercising a stimulating effect on other plants. The volatile monoterpenoids camphor and 1,8-cineole are considered to be responsible for the well-known “*Salvia* phenomenon” in natural settings, explained by their inhibitory effects on surrounding species. Further evidence for allelopathic effects exists for genera such as *Clinopodium [Calamintha]*, *Coleus*, *Conradina*, *Dracocephalum [Hyssopus]*, *Hyptis*, *Lavandula*, *Leonurus*, *Leucas*, *Mentha*, *Nepeta*, *Ocimum, Origanum*, *Orthosiphon*, *Salvia*, *Satureja*, *Tectona*, and *Thymus*. But most studies investigated the allelopathic effect of certain isolated extracts or compounds [147], hence leaving a gap in the literature on the effect of volatiles that are only emitted in the air by plants. Out of the wide range of potential agricultural interests in these mechanisms, the phytotoxicity of volatiles released by aromatic plants against noxious weeds is of greatest importance—for weed control as natural bioherbicides [148]. Although the VOC-mediated allelopathy mechanisms at large still remain poorly documented and insufficiently understood [149], perhaps a good starting point for future research is to identify the taxonomic groups that might present the best potential. In this sense, arguments from this work sustain the idea that Lamiaceae is a good candidate for further study.

Regarding the effects of plants from the family Lamiaceae in intercropping schemes, these are not always so easily defined. A study that intercropped *Salvia officinalis* with *Vitis vinifera* “Trebbiano Romagnolo” did not find changes in the vineyard productivity parameters. But, more interestingly, the volatiles emitted by sage interacted with the grapevines and influenced the accumulation of volatile compounds. Particularly, eugenol in its bound form increased in grape berries. It was proposed that this could be due to the effect of VOCs produced by sage plants on biosynthesis pathways in grape berries [150].

In addition to applications in agriculture, aromatic species from this botanic family can find other applications right in the cities. Plant species such as *Lavandula angustifolia*, *Mentha longifolia*, *Salvia officinalis*, *Salvia rosmarinus*, and *Thymus vulgaris* have been suggested as suitable for sensory-therapeutic gardens. Notably, these provide olfactive and therapeutic value to specific landscaping settings [151].

The aromatic species of the family Lamiaceae were shown to emit volatile compounds (most often monoterpenes), and due to this fact, these can find in situ usefulness before they are harvested and processed. Thus, here we presented preliminary evidence of their beneficial use in agriculture (intercropping) and landscaping. Therefore, both in rural as well as urban spaces, species from the family Lamiaceae can play roles that spring up from the aerobiology relevance of their volatiles, and we propose further study of these applications.

## 5. Concluding Overview

Lamiaceae is a large family of flowering plants, comprising many of the aromatic species that are economically important for many industries. Despite its widespread presence, expanding cultivation, and use, there is no current overview of the aerobiology of this family. Hence, the aim of this work is to provide a general view and novelties on the aerobiology of the family Lamiaceae. It was identified that bioaerosols of interest in this family are, firstly, the abundant volatile organic compounds released in the air by the plants and to a lesser extent the pollen.

Although pollen is a large chapter of aerobiology, the flower morphology in Lamiaceae is specialized for pollinator-mediated transfer of pollen, a fact also evident due to the reduced number of stamens and quantity of pollen produced. The low probability of pollen presence in the air from these species might be due to this. This further suggests that the probability of pollen allergy might remain low even upon the mass flowering of these species.

Volatile organic compounds are the biogenic aerosols of greatest importance for the aerobiology of this species. The sites of biosynthesis of volatiles emitted by aromatic species are the glandular trichomes at the surface of the plants. Based on general morphology, two main types have been described in the Lamiaceae: peltate trichomes with a disk-shaped head and very short stalk and capitate with a longer stalk and head comprising one to several cells. Literature research indicated that volatile emissions of Lamiaceae species are abundant in monoterpenes, sesquiterpenes, and diterpenes. The most important monoterpenes reported across studies were α-pinene, β-pinene, 1,8-cineole, menthol, limonene, and γ-terpinene, and the most frequent sesquiterpenes were γ-muurolene, β-caryophyllen, and germacrene D. Because diterpenes and triterpenes are less volatile and less abundant, these are less significant components in volatile emission from fresh plants.

The interaction of organic particles and molecules released by the plants and their environment suggests at least some aromatic species could be used for beneficial results. This represents the novel dimension of the aerobiology applications for these species. Based on emerging evidence, it is recommended to include aromatic species of Lamiaceae in intercropping schemes to enhance yields and the health of the crops. Also, farmers could consider these species in the planning of the crop structure in a field, to take advantage of natural pest control and enhanced yields.

## Figures and Tables

**Table 1:** Synopsis of major volatile compounds emissions reported in some Lamiaceae species.

Species	Class *	Compound	Source
*Ajuga iva* (L.) Schreb.	MHs	α-pinene, β-pinene, limonene	[81]
	OMs	linalool	[81]
*Agastache mexicana* (Kunth) Lint & Epling	MHs	myrcene	[82]
	OMs	α-citral, β-citral, citronellol, geraniol, geranyl acetate	[82]
*Agastache aurantiaca* (A.Gray) Lint & Epling	OMs	menthone, pulegone	[82]
	SHs	β-caryophyllene	[82]
*Betonica macrantha* K.Koch	MHs	α-pinene, p-cymene	[83]
	OMs	carvacrol, thymoquinone, methyl ether	[83]
	SHs	(E)-caryophyllene	[83]
*Coleus amboinicus* Lour.	MHs	o-cymene, γ-terpinene	[84]
	OMs	carvacrol	[84]
	SHs	(E)-caryophyllene, α-*trans*-bergamotene	[84]
*Coleus comosus* Hochst. ex Gürke	MHs	α-thujene, α-pinene	[85]
	SHs	β-caryophyllene, germacrene-D, *trans*-β-guaiene	[85]
	NTs	1-octen-3-ol	[85]
*Clinopodium candidissimum* (Munby) Kuntze	OMs	isopulegone, pulegone, neo-menthol, piperitenone	[86]
	SHs	bicyclogermacrene, germacrene D	[86]
*Clinopodium nepeta* (L.) Kuntze	MHs	α-pinene, β-pinene, limonene	[87]
	OMs	piperitone oxide	[87]
	SHs	caryophyllene, germacrene D	[87]
*Clinopodium pulegium* (Rochel) Bräuchler	MHs	α-pinene, β-pinene	[79]
	OMs	menthone, pulegone, isomenthone	[79]
*Clinopodium rouyanum* (Briq.) Govaerts	MHs	limonene	[88]
	OMs	menthone, pulegone	[88]
	SHs	germacrene-D	[88]
*Dracocephalum kotschyi* Boiss.	MHs	limonene	[89]
	OMs	geranial, limonene-10-al	[89]
	NTs	1,1-dimethoxy decane	[89]
*Dracocephalum officinalis* (L.) Y.P.Chen & B.T.Drew	MHs	sabinene, β-pinene	[90]
	OMs	*cis*-pinocamphone, terpinen-4-ol, carvacrol	[90]
*Elsholtzia splendens* Nakai ex F.Maek.	OMs	naginata ketone, geranyl acetate, dehydro carveol	[91]
*Lamium amplexicaule* L.	MHs	α-pinene	
	OMs	*trans*-chrysanthenyl acetate	[92]
	SHs	germacrene D, β-caryophyllene	[92]
	NTs	(E)-3-hexenyl acetate	
*Lamium bifidum* Cirillo	MHs	myrcene, sabinene, α-pinene, (Z)-ocimene	[92]
	SHs	β-caryophyllene, germacrene D, α-humulene, β-elemene	[92]
*Lamium hybridum* Vill.	MHs	α-pinene, β-pinene, (Z)-ocimene	[92]
	SHs	β-caryophyllene, germacrene D, (E) β-farnesene, bicyclogermacrene	[92]
*Lamium purpureum* L.	MHs	α-pinene, β-pinene	[92]
	SHs	germacrene D, β-elemene, valencene, (E) β-farnesene	[92]
	NTs	(E)-3-hexenyl acetate, n-pentadecane, decanal, (E)-2-decenal	[92]
*Lavandula angustifolia* Mill.	MHs	α-pinene, δ-3-carene, (Z) β-ocimene,	[87]
	MHs	myrcene	[90]
	OMs	camphor, 1,8-cineol	[87]
	OMs	linalool, α-terpineol, linalyl acetate, lavandulyl acetate, caryophyllene	[90]
	SHs	caryophyllene, *trans*-γ-cadinene, germacrene D, *trans*-α-bergamotene	[87]
*Lavandula viridis* L’Hér.	OMs	camphor, 1,8-cineol	[93]
*Marrubium anisodon* K.Koch	MHs	myrcene, α-phellandrene, p-cymene, limonene, *trans*-β-ocimene	[94]
	OMs	linalool	[94]
	SHs	β-caryophyllene	[94]
*Marrubium persicum* C.A.Mey.	MHs	α-thujene, *trans*-β-ocimene, α-terpinolene	[95]
	OMs	camphor	[95]
	SHs	caryophyllene, δ-elemene, β-elemene	[95]
	OSs	caryophyllene oxide	[95]
	PPs	eugenol, methyl eugenol	[95]
*Micromeria inodora* (Desf.) Benth.	OMs	1,8-cineole, camphor, α-terpinyl acetate	[96]
	SHs	β-caryophyllene, allo-aromadendrene	[96]
*Melissa officinalis* L.	MHs	(Z)-b-ocimene	[97]
	OMs	citronellal, citronellol, (E)-citral, (Z)-citral, geraniol	[97]
	SHs	β-caryophyllene	[97]
*Melittis melissophyllum* L. subsp. *melissophyllum*	MHs	α-pinene	[98]
	OMs	*cis*-chrysanthenyl acetate	[98]
	NTs	1-octen-3-ol, coumarin	[98]
*Mentha arvensis* L. ‘Thai’	MHs	limonene	[99]
	OMs	carvone	[99]
*Mentha haplocalyx var. piperascens* (Malinv. ex Holmes) C.Y.Wu & H.W.Li	MHs	limonene	[99]
	OMs	p-menthone, menthol, menthofuran, neomenthyl acetate	[99]
*Mentha × piperita* L.	MHs	D limonene	[66]
	OMs	D-carvone, eucalyptol	[66]
	OMs	1,8-cineole, p-menthone, menthol, menthofuran, neomenthol, neomenthyl acetate	[99]
	SHs	(+)-epi-bicyclo sesquiphellandren	[66]
*Mentha pulegium* L.	MHs	limonene, sabinene	[100]
	OMs	1,8-cineole, carvone	[100]
	ODs	methyl neoabietate	[100]
	NTs	oleic acid, n-hexadecanoic acid, hexacosane, heptacosane, α-monoolein	[100]
*Mentha spicata* L.	OMs	eucalyptol	[66]
	SHs	caryophyllene, D-germacrene, manoyl oxide, γ-muurolene	[66]
	MPRs	2,5-dimethyl-3 methoxypyrazine	[66]
*Mentha suaveolens* Ehrh.	OMs	*trans*-piperitenone oxide	[99]
	SHs	germacrene D	[99]
*Nepeta cataria* L.	OMs	β-citronellol, nerol, geraniol, α-citral	[90]
*Nepeta × faassenii* Bergmans ex Stearn ‘Six Hills Giant’	OMs	*cis*-*trans*-nepetalactone	[64]
	SHs	β-caryophyllene, germacrene D	[101]
*Ocimum basilicum* L.	MHs	D-limonene, β-myrcene	[102]
	OMs	1,8-cineole, linalool, bornyl acetate, estragole (methyl chavicol)	[102]
	SHs	bergamotene, β-muurolene	[102]
	PPs	eugenol	[102]
*Ocimum basilicum* L. ‘Blue Spice’	MHs	(E)-β-ocimeme	[101]
	SHs	*trans*-α-bergamotene, germacrene D, β-bisabolene, *trans*-α-bisabolene, β-caryophyllene	[101]
	PPs	eugenol	[101]
*Ocimum basilicum* L. ‘Cinnamon’	OMs	linalool	[101]
	SHs	β-elemene, α-guaiene, germacrene D, β-bulnesene	[101]
	PPs	eugenol	[101]
*Origanum acutidens* (Hand.-Mazz.) Ietsw.	MHs	p-cymene, γ-terpinene	[103]
	OMs	carvacrol, thymol	[103]
*Origanum vulgare* subsp. *gracile* (K.Koch) Ietsw.	MHs	γ-terpinene	[103]
	OMs	carvacrol, thymol	[103]
*Phlomis grandiflora* H.S.Thomps. var. *grandiflora*	MHs	α-pinene, limonene	[104]
	SHs	α-cedrene, β-caryophyllene, germacrene D, α-curcumene	[104]
*Phlomis monocephala* P.H.Davis	MHs	α-pinene	[105]
	SHs	(E)-β-farnesene, germacrene D, bicyclogermacrene, δ-cadinene, α-cubebene	[105]
	NTs	3-Octen-2-one	[105]
*Phlomis rigida* Labill.	SHs	β-caryophyllene, germacrene D	[105]
	NTs	(E)-2-hexenal	[105]
*Pseudodictamnus acetabulosus* (L.) Salmaki & Siadati	SHs	β-caryophyllene, γ-muurolene, (E)-β-ionone, aromadendrene, γ-elemene	[106]
	OSs	(E)-nerolidol	[106]
	NTs	1-decanal, 1-nonanal	[106]
	ACs	(E)-geranyl acetone	[106]
*Salvia rosmarinus* Spenn.	MHs	α-pinene, camphene, β-pinene	[87,107,108]
	MHs	limonene, P-cymene	[107]
	MHs	myrcene	[87]
	OMs	borneol, bornyl acetate, 1,8-cineol	[87]
	OMs	camphor	[107,108]
	SHs	α-copaene, trans-caryophyllene, α-amorphene	[107]
	SHs	caryophyllene, α-humulene	[87]
*Salvia aethiopis* L.	MHs	α-pinene, β-pinene, camphene, sabinene, (E)-β-ocimeme	[109]
	SHs	alloaromadendrene, β-caryophyllene	[109]
	ACs	(E)-geranyl acetone	[109]
*Salvia amplexicaulis* Lam.	MHs	β-pinene, limonene	[109]
	SHs	β-caryophyllene, germacrene D, α-gurjunene, alloaromadendrene, valencene	[109]
*Salvia austriaca* Jacq.	MHs	α-pinene, β-pinene, limonene, (Z)-β-ocimeme	[109]
	OMs	α-thujone	[109]
	NTs	n-octanol, n-pentadecane	[109]
*Salvia blepharophylla* Brandegee ex Epling	OMs	1,8-cineole, methyl carvacrol, isobornyl formate, linalool	[110]
	SHs	β-bourbonene, β-caryophyllene	[110]
	OSs	(Z)-sesquilavandulol	[110]
*Salvia dentata* Aiton	MHs	α-pinene, camphene, β-pinene, δ-3-Carene,	[111]
	OMs	1,8-cineole, camphor, bornyl acetate	[111]
*Salvia discolor* Kunth	SHs	β-caryophyllene, α-humulene, β-elemene, β-bisabolene	[101]
	OSs	elemol acetate	[101]
	ODs	methyl neoabietate	[101]
*Salvia elegans* Vahl	SHs	β-caryophyllene, germacrene D, valencene, α-gurjunene, α-humulene	[109]
*Salvia greggii* A.Gray	MHs	β-pinene, limonene	[110]
	OMs	1,8-cineole	[110]
	SHs	γ-muurolene, β-caryophyllene, β-gurjunene, germacrene D	[110]
*Salvia microphylla* Kunth ‘Hot Lips’	MHs	p-cymene, limonene, γ-terpinene, eucalyptol	[101]
	OMs	isobornyl acetate, camphor	[101]
	SHs	α-copaene	[101]
	OSs	guaiol, cubebol	[101]
*Salvia officinalis* L.	MHs	α-pinene, β-pinene, camphene, β-myrcene	[107]
	MHs	α-pinene, camphene, β-pinene	[109]
	OMs	1,8-cineole, α-thujone, β-thujone, camphor	[107,109,112]
	SHs	*trans*-caryophyllene	[107]
*Salvia officinalis* L. ‘Purpurascens’	OMs	β-thujone	[109]
	SHs	α-humulene	[109]
	OSs	globulol	[109]
	NTs	isoamyl dodecanoate, n-hexadecane	[109]
*Salvia scabra* Thunb.	OMs	1,8-cineole, camphor	[111]
	SHs	α-copaene, β-caryophyllene, β-copaene, germacrene D, 9-epi-(E)-caryophyllene, γ-elemene	[111]
	OSs	nuciferol acetate	[111]
	DHs	cembrene, isopimara-9(11),15-diene	[111]
*Salvia sclarea* L.	MHs	α-pinene	[90]
	OMs	linalool acetate, linalool	[113]
	OMs	α-terpineol, linalyl acetate, linalool	[90]
*Salvia splendens* Sellow ex Nees	NTs	isopropyl tetradecanoate	[109]
*Salvia transsylvanica* (Schur ex Griseb. & Schenk) Schur	SHs	β-caryophyllene	[109]
	OSs	(E)-nerolidol, acorenone, (E,E)-farnesyl acetate	[109]
	ACs	(E)-geranyl acetone	[109]
	NTs	isopropyl tetradecanoate, n-decanal, isoamyl dodecanoate, n-heptadecane, n-octadecane	[109]
*Salvia verticillata* L.	OMs	1,8-cineole	[114]
	SHs	δ-elemene; β-caryophyllene, germacrene D, γ-muurolene, bicyclogermacrene, β-bourbonene, β-copaene, α-humulene, (E)-β-farnesene	[114]
*Satureja hortensis* L.	MHs	α-thujene, α-pinene, myrcene, β-pinene, α-terpinene, p-cymene, γ-terpinene	[87,115]
	OMs	methyl thymol, methyl carvacrol, thymol	[87]
	SHs	caryophyllene, β-bisabolene	[87]
*Scutellaria altissima* L.	MHs	(E)-β-ocimene, (Z)-β-ocimene	[116]
	SHs	γ-muurolene	[116]
	ACs	(E)-geranyl acetone	[116]
	NTs	(Z)-3-hexenol acetate, (E)-3-hexen-1-ol, decanal	[116]
*Scutellaria baicalensis* Georgi	SHs	β-caryophyllene, germacrene D, δ-cadinene, γ-muurolene, γ-cadinene, α-humulene, α-copaene, α-muurolene, bicyclogermacrene	[117]
*Scutellaria caucasica* A.Ham.	OMs	1,8-cineole	[118]
	SHs	γ-muurolene, β-caryophyllen, germacrene D, α-humulene	[118]
*Scutellaria californica* A.Gray	SHs	β-caryophyllene, germacrene D, β-bourbonene	[119]
	NTs	methyl 2-methylbutyrate, methyl butyrate	[119]
*Sideritis condensata* Boiss. & Heldr.	MHs	α-pinene, β-pinene, limonene, β-ocimene	[120]
	SHs	germacrene-D, *trans*-caryophyllene, (E)-β-farnesene, α-copaene, bicyclogermacrene	[120]
	NTs	cuminaldehyde	[120]
*Sideritis montana* L.	MHs	limonene	[121]
	SHs	*trans*-caryophyllene, *trans*-β-farnesene, germacrene D, bicyclogermacrene, γ-cadinene, δ-cadinene, α-amorphene	[121]
*Sideritis romana* L.	MHs	β-pinene, limonene	[121]
	SHs	allo aromadendrene, *trans*-β-farnesene, isocaryophyllene, bicyclogermacrene	[121]
*Stachys annua* L. subsp. *annua* var. *annua*	MHs	α-pinene, β-pinene, (Z)-β-ocimene	[83]
	SHs	β-bourbonene	[83]
*Stachys lavandulifolia* Vahl	MHs	myrcene, α-pinene, β-phellandrene, β-pinene, α-phellandrene, sabinene	[122]
*Stachys sylvatica* L.	MHs	limonene, α-pinene, sabinene	[83]
	SHs	γ-muurolene, α-cedrene, α-humulene, α-zingiberene	[83]
	NTs	(2E)-hexenal	[83]
*Teucrium chamaedrys* L.	OMs	1,8-cineole	[123]
	SHs	β-cubebene, α-cubebene, β-caryophyllene, α-humulene,γ-muurolene, β-bourbonene, (E)-β-farnesene	[123]
*Teucrium marum* L.	OMs	dolichodial, dolicholactone, estragole	[124]
	SHs	(E)-α-bergamotene, (E)-β-caryophyllene, β-bisabolene, β-sesquiphellandrene	[124]
*Teucrium polium* L.	SHs	β-farnesene, *trans*-caryophyllene, γ-elemene, allo-aromadendrene, α-guaiene, α-gurjunene, δ-guaiene	[125]
	OSs	farnesol	[125]
	ACs	geranyl acetone	[125]
	NTs	6-methyl-5-hepten-2-one	[125]
*Thymus algeriensis* Boiss. and Reut.	MHs	α-pinene, camphene, β-myrcene	[125]
	OMs	camphor, linalyl acetate, bornyl acetate, 1,8-cineole, α-terpineol, borneol	[125]
	SHs	β-farnesene	[125]
*Thymus serpyllum* L.	MHs	p-cymene, γ-terpinene	[107]
	OMs	carvacrol methyl ether, thymol methyl ether	[107]
	SHs	β-bisabolene, *trans*-caryophyllene	[107]
*Thymus vulgaris* L.	MHs	p-cymene, γ-terpinene	[126]
	OMs	thymol, carvacrol	[126]
	SHs	β-caryophyllene	[126]
*Ziziphora capitata* L.	MHs	α–pinene, β–Pinene, limonene, p–mentha–3,8–diene, (Z)–b–ocimene	[127]
	OMs	p–menth–3–en–8–ol, pulegone	[127]

* Note: Terpenoids: monoterpene hydrocarbons (MHs); oxygenated monoterpenes (OMs); sesquiterpene hydrocarbons (SHs); oxygenated sesquiterpenes (OSs); diterpene hydrocarbons (DHs); oxygenated diterpenes (ODs); triterpenes hydrocarbons (THs); other: methoxypyrazines (MPRs), phenylpropanoids (PPs), non-terpene derivatives (NTs), apocarotenoids (ACs). Volatile compounds identified by headspace solid-phase microextraction technique from aerial parts of plants, which account for more than 3% from total constituents. Species list according to Plants of the World Online [128].

## Data Availability

No new data were created or analyzed in this study.
